# Family Size and Past Obstetric History of Married Women Aged 45 Years and Older in Erbil

**DOI:** 10.7759/cureus.73890

**Published:** 2024-11-18

**Authors:** Halbeen A Adil, Namir Al-Tawil

**Affiliations:** 1 Community Medicine, Kurdistan Higher Council of Medical Specialties, Erbil, IRQ; 2 Community Medicine, Hawler Medical University, Erbil, IRQ

**Keywords:** adolescent pregnancy, cesarean section rate, early marriage, family size, large family, lifetime fertility rate, preferred family size, short birth-spacing interval, socio-economical status, unplanned pregnancies

## Abstract

Background and aims: The family size plays an important role in fulfilling the primary needs of each family member and providing a quality life for them. This research studies the association of family size with various socioeconomic and demographic factors and the lifetime fertility patterns of previous decades.

Methods: This cross-sectional study was conducted at the Maternity Teaching Hospital in Erbil, Kurdistan Region of Iraq (KRI). The data was collected from December 11, 2023, to April 15, 2024, after the ethics committee of the Kurdistan Higher Council for Medical Specialties approved the proposal. A convenience sample of 400 women aged 45-74 years who had completed their families were directly interviewed. The data was analyzed using IBM SPSS Statistics for Windows, Version 26 (Released 2019; IBM Corp., Armonk, New York, United States).

Results: The prevalence of large family size (seven members and above) was 78.5%. The lifetime fertility rate of the women was 6.78 live births per woman. Large family size is significantly (p < 0.001) associated with age <18 years at marriage, short birth spacing interval, and unplanned pregnancies. The majority (91.7%) of the participants were grand multipara. The Cesarean section (CS) rate was 7.3%, 60.2% of the births were delivered at home, and 46.6% of the live births were delivered with a preceding birth interval of less than two years. The majority (82.2%) of the pregnancies were unplanned.

Conclusions: Large family size is highly prevalent among women who have completed their families in Erbil. Many demographic and socioeconomic factors are associated with large family sizes, such as rural residency, age <18 years at marriage, low socioeconomic status (SES), short birth spacing interval, and unplanned pregnancies. For a more desired family size, it is recommended that young females be empowered by encouraging their education and providing occupation opportunities. In addition to that, prohibiting early marriage and providing/strengthening premarital programs focusing on the importance of family planning including birth spacing and utilizing contraceptive methods properly play a great role.

## Introduction

The family size plays an important role in fulfilling the primary needs of each family member and providing a quality life for them [1]. Family size, the number of children born to a woman with the parents [2], is categorized into small family (up to three members), medium family (four to six members), and large family (seven members and above) [3].

Early marriage (<18 years) and adolescent birth (mother aged <20 years) are more common in larger families [4], while higher intelligence quotient (IQ) [5], academic accomplishment, and professional success are typically associated with smaller households [6]. Mothers having big families are more likely to suffer from several illnesses like hypertension, diabetes, gall bladder problems, and cervical cancer [4,7]. High parity and advanced maternal age are significantly associated with higher maternal [8] and perinatal mortality rates [8,9]. Despite the risks of high parity, it is worth mentioning that there is an inverse association between parity and the risk of some cancers like endometrial cancer [10], ovarian cancer [11], and breast cancer [12].

Although the total fertility rate (TFR) of Iraq is declining [13], it is still the highest among the neighboring countries [14]. In 2022, the TFR of Iraq, Jordan, Syria, Turkey, and Iran were 3.4, 2.8, 2.7, 1.9, and 1.7 births per woman, respectively [14]. Compared to the central and south of Iraq, the Kurdistan Region had a lower rate of 3.2 [13]. In Erbil, the TFR was 3.1 births per woman [15]. The average number of ever-born children (lifetime fertility) of Iraqi women by the end of their reproductive life (age group: 45-49 years) was 6.38 in 2006 [16].

Early marriage, adolescent birth, short birth interval between consecutive births, and low rate of contraceptive use influence fertility rate and are associated with increased rates of unplanned pregnancies, induced abortions, and maternal and infant mortality [11,17]. When the interval between two consecutive live births is less than two years, the infant mortality rate is 45% higher than when the interval is two to three years and 60% higher when it is four years or more [18]. Nonetheless, the contraceptive prevalence rate in Erbil was 66% for all methods and 24.9% for modern methods according to the Multiple Indicator Cluster Surveys-6 (MICS6) [15].

We conducted this research to study the association between family size and various socioeconomic and demographic factors as well as the lifetime fertility patterns of the previous decades. To the extent of our knowledge, there is no original article on this topic specific to Erbil. Documenting these findings will benefit in identifying gaps which consequently might pave the way for shaping policies and programs that aim at achieving Sustainable Development Goals (SDGs) for better health.

The objectives of the study were to assess the family size, proportion of large-size families, and factors associated with large-size families; to find out the outcome of pregnancies and their mode of delivery; to identify the percentage of births with short birth spacing intervals; and lastly, to find out the contraceptive use rate and the proportion of unplanned pregnancies.

## Materials and methods

This cross-sectional study was conducted at the Maternity Teaching Hospital in Erbil City, the capital of the Kurdistan Region of Iraq (KRI). The data were collected from December 11, 2023, to April 15, 2024. The Kurdistan Higher Council for Medical Specialties Ethics Committee approved the research proposal on November 28, 2023 (approval no. 1974). Epi Info 7.2 (Centers for Disease Control and Prevention (CDC); Atlanta, Georgia, USA) was used to determine the sample size, setting the alpha at 0.05, the estimated prevalence of large-size families at 50% (as it gives the largest sample size) [19], and the absolute precision at 5%. The estimated sample size was 384; a 5% nonresponse rate was expected, corresponding to 20 participants; therefore, 404 women were included. Four of those women refused to participate; so 400 women were studied which was a 99% response rate. The convenience sampling method was used, and any woman (from the relatives of the patients) aged 45-74 years who visited the hospital (during the presence of the researcher) and met the inclusion criteria was included in the study.

The eligible respondents were married women aged 45-74 years who had completed their families. The exclusion criteria included any of the following: widowed, divorced, separated, those with no children, those with mental and memory impairment, and those who had undergone hysterectomy before reaching 45 years.

Those women were directly interviewed after obtaining their verbal consent, and the questionnaires were filled out using Google Forms. The questionnaire designed by the researchers was used for data collection, and each interview lasted an average of 20 minutes. The direct interview method was chosen because a significant proportion of women in this age group (45-74 years) are illiterate [20], making it difficult for them to use technology, and some questions required additional clarification, such as those regarding gravidity, parity, miscarriages, etc. Before data collection, a pilot study was conducted with 20 women, after which necessary adjustments to the questionnaire were made accordingly. Those 20 women were not included in the main study.

The questionnaire consisted of two parts; part one contained questions regarding the sociodemographic data of the participants. A 21-point scoring system, adapted from Shabu and Al-Tawil [21], was used to classify the women according to their socioeconomic status (SES). The total score (21) was divided into three equal categories: one­­ to seven (low), eight to 14 (medium), and 15-21 (high). Seven variables were used: husband education (four scores), wife education (four scores), husband occupation (four scores), wife occupation (four scores), house ownership (two scores), car ownership (one score), and income (two scores). The second part included data on pregnancies and deliveries; in addition to other questions related to past obstetric history, it also included questions regarding family size preference, birth spacing, unplanned pregnancies, contraceptive use, etc.

Early marriage was defined as a marriage in which one or both spouses are under 18 years old. "The age at marriage," used in the current study, was meant by "the age at first marriage." Teenage pregnancy or adolescent pregnancy was regarded as pregnancy among girls under the age of 20 years [22]. The household was defined as a social unit composed of those living together in the same dwelling [23]. We have classified family status into a nuclear family, a family consisting of parents with their children, and an extended family, consisting of parents, their children, grandparents, and/or other relatives [24]. The completed (lifetime) fertility rate is the average number of ever-born live births to women during their entire reproductive life [25]. Gravidity (G) is the number of pregnancies a woman has had regardless of the outcome of pregnancy. Primigravida, multigravida, and grand multigravida refer to G1, G2-4, and G5+, respectively. Parity (P) is the number of deliveries, both live births and stillbirths, a woman has had, that have reached viability (24 weeks gestation). Primipara, multipara, and grand multipara refer to P1, P2-4, and P5+, respectively [26,27]. Miscarriage (spontaneous abortion) is defined as pregnancy loss before reaching 24 weeks gestation (for respondents’ better understanding we used "less than six months" to be regarded as miscarriage). Live birth is the birth of a child that showed any sign of life, while stillbirth refers to the death of a fetus after 24 weeks of gestation [26]. Living children are the number of currently living children a woman has [27]. The number of babies in the "mode of delivery" and "place of delivery" sections in our study included both live births and stillbirths.

Unplanned pregnancy is the occurrence of pregnancy at an undesired time or when no more children are wanted [28]. Short birth spacing is the interval between two consecutive live births that is less than 24 months [29].

Family size was calculated as the number of parents (two) and the currently living children, even if they were not living with the family for the time being. Large-sized families were defined as those having five or more children (in addition to the parents) [3]. However, the household size was defined as the number of all members of a dwelling, who live together in the same house at the time of the survey. The crowding index was calculated as the number of households per the number of rooms except kitchens and bathrooms [30].

As traditional (natural) contraceptive methods, we have included safe period, body temperature, withdrawal, and lactation. On the other hand, modern contraceptive methods (depending on the available commodities) included male condoms, female condoms, spermicides, oral contraceptive pills, injections, implants, intrauterine contraceptive device (IUCD), tubal ligation, and vasectomy.

Regarding the data analysis, the IBM SPSS Statistics for Windows, Version 26 (Released 2019; IBM Corp., Armonk, New York, United States) was used, and a p-value of ≤0.05 was regarded as statistically significant. Descriptive statistics, such as mean and standard deviation, were used for numerical variables and frequency and percentage for categorical variables. Moreover, the Chi-square test was used for the association between two categorical variables, and Fisher’s exact test was used when the expected count of more than 20% of the cells of the table was less than five. Lastly, the logistic regression analysis was used to control for confounding factors and predict the outcome, which was the large family size. 

## Results

The total number of women invited to participate in the study was 404. Only four cases refused participation, so the nonresponse rate was 1%. The mean age (SD) of women was 52.3 (6.1) years. The majority of the respondents were Kurds (81.8%), and almost all were Muslims (97.3%). The majority (85%) of the women were living in urban areas, and 42.0% of them were living inside Erbil City. Nearly half (43.3%) of the women married at the age of less than 18 years, while 46.8% of their husbands were married at ages 18-24 years. More than half (55.0%) of the families were nuclear.

The prevalence of large family size was 78.5%. The lifetime (completed) fertility rate of the women was 6.78 live births per woman. Table 1 shows the family size by sociodemographic characteristics. The differences between the percentages of large-size families among the age groups were statistically insignificant (p = 0.301). However, the highest percentage (88.0%) was seen in the eldest age group (65 and more), and the lowest (73.5%) was seen in the youngest age group (45-49). In contrast to that, it was highly significant (p < 0.001) between groups of geographical area, ethnicity, religion, and family status. It was more prominent in rural areas than in urban areas. It was higher in the periphery of Erbil City (88.7%) and the villages (88.3%) than the places located inside Erbil City (66.7%), districts, and subdistricts of Erbil City (77.4%). The majority, 91.8%, of Arab participants were having large families, compared with 79.8% among Kurds. The proportion of large families was much higher among Muslims (80.2%) than Christians (18.2%). The relationship between the age of both wife and husband at marriage and large family size was inversely proportional; the younger the age at marriage, the higher the prevalence of large family size (p < 0.001). The mean ages of the wife and husband at marriage were 19 (min: 11) and 24 (min: 12) years, respectively. Around half (55.3%) of the women had their first pregnancy at ages less than 20 years, among which 90.0% had large family size (p < 0.001); the mean age of respondents at the first pregnancy was 19.9 years. The prevalence of large family size in the extended families (87.8%) was significantly (p < 0.001) higher than that of the nuclear families (70.9%) (Table 1). The average household size was 7.3 members per household.

**Table 1:** Family size by sociodemographic characteristics *Column total was calculated. **p-value was calculated by Pearson Chi-Squared test. ***p-value was calculated by the Fisher’s exact test

Variables	Large family size	Small/medium family size	Total	p-value	Statistical test value
	No. (%)	No. (%)	No. (%) *		
Age				0.301**	4.868**
45-49	108 (73.5)	39 (26.5)	147 (36.8)		
50-54	114 (80.3)	28 (19.7)	142 (35.5)		
55-59	43 (84.3)	8 (15.7)	51 (12.8)		
60-64	27 (77.1)	8 (22.9)	35 (8.8)		
65 and more	22 (88.0)	3 (12.0)	25 (6.3)		
Geographic area				<0.001**	26.008**
Inside Erbil City	112 (66.7)	56 (33.3)	168 (42.0)		
Periphery of Erbil City	125 (88.7)	16 (11.3)	141 (35.3)		
Districts and subdistricts of Erbil City	24 (77.4)	7 (22.6)	31 (7.8)		
Villages	53 (88.3)	7 (11.7)	60 (15.0)		
Residency				0.040**	4.044**
Urban	261 (76.8)	79 (23.2)	340 (85.0)		
Rural	53 (88.3)	7 (11.7)	60 (15.0)		
Religion				<0.001***	24.383***
Muslim	312 (80.2)	77 (19.8)	389 (97.3)		
Christian	2 (18.2)	9 (81.8)	11 (2.8)		
Ethnic group				<0.001***	30.971***
Kurd	261 (79.8)	66 (20.2)	327 (81.8)		
Turkmen	6 (46.2)	7 (53.8)	13 (3.3)		
Arab	45 (91.8)	4 (8.2)	49 (12.3)		
Chaldean and Assyrian	2 (18.2)	9 (81.8)	11 (2.8)		
Age of wife at marriage				<0.001**	86.215**
< 15	56 (90.3)	6 (9.7)	62 (15.5)		
15-17	101 (91.0)	10 (9.0)	111 (27.8)		
18-20	96 (87.3)	14 (12.7)	110 (27.5)		
21-23	30 (63.8)	17 (36.2)	47 (11.8)		
24-26	23 (59.0)	16 (41.0)	39 (9.8)		
≥27	8 (25.8)	23 (74.2)	31 (7.8)		
Age of wife at first pregnancy				<0.001**	39.003**
<20	199 (90.0)	22 (10.0)	221 (55.3)		
≥20	115 (64.2)	64 (35.8)	179 (44.7)		
Age of husband at marriage				<0.001**	30.429**
<18	46 (92.0)	4 (8.0)	50 (12.5)		
18-24	162 (86.6)	25 (13.4)	187 (46.8)		
25-34	88 (65.7)	46 (34.3)	134 (33.5)		
≥35	18 (62.1)	11 (37.9)	29 (7.3)		
Family status				<0.001**	16.691**
Nuclear	156 (70.9)	64 (29.1)	220 (55.0)		
Extended	158 (87.8)	22 (12.2)	180 (45.0)		
Total	314 (78.5)	86 (21.5)	400 (100)		

Table 2 is about the family size by socioeconomic characteristics. It shows that 44.5% of respondents were illiterate (literacy rate: 55.5%), and from those, 89.3% had a large family size. It shows that the higher the wife's education level, the lower the prevalence of large families (p < 0.001). Regarding husbands’ education, the largest proportion of the husbands (39.8%) can read and write or were of primary education. In general, low rates of large families were found among educated husbands (p < 0.001). The majority (87.5%) of respondents were housewives, while 39% of husbands were unskilled manual workers. The association between the occupation of wife and husband and large family size was highly significant (p < 0.001). The higher the rank of the occupation, the less the prevalence of large families. The majority (88.3%) of women with low SES had large families, compared with 26.5% of women with high SES (p < 0.001), as presented in Table 2.

**Table 2:** Family size by socioeconomic characteristics *Column total was calculated. **p-value was calculated by Pearson Chi-squared test.  ***p-value was calculated by Fisher’s exact test

Variables	Large family size	Small/medium family size	Total		p-value	Statistical test value
	No. (%)	No. (%)	No. (%) *			
The educational level of the wife				<0.001**		69.618**
Illiterate	159 (89.3)	19 (10.7)	178 (44.5)			
Read and write or primary	127 (79.9)	32 (20.1)	159 (39.8)			
Intermediate	18 (64.3)	10 (35.7)	28 (7.0)			
Secondary	6 (40.0)	9 (60.0)	15 (3.8)			
College and above	4 (20.0)	16 (80.0)	20 (5.0)			
The educational level of the husband				<0.001**		35.819**
Illiterate	78 (85.7)	13 (14.3)	91 (22.8)			
Read and write and primary	141 (88.7)	18 (11.3)	159 (39.8)			
Intermediate	49 (69.0)	22 (31.0)	71 (17.8)			
Secondary	23 (60.5)	15 (39.5)	38 (9.5)			
College and above	23 (56.1)	18 (43.9)	41 (10.3)			
Wife occupation				<0.001***		72.768***
Housewife	297 (84.9)	53 (15.1)	350 (87.5)			
Unskilled manual worker	10 (83.3)	2 (16.7)	12 (3.0)			
Skilled manual worker	1 (50.0)	1 (50.0)	2 (0.5)			
Nonmanual worker	5 (17.9)	23 (82.1)	28 (7.0)			
High-level occupations	1 (12.5)	7 (87.5)	8 (2.0)			
Husband occupation					<0.001**	34.204**
Unemployed	68 (90.7)	7 (9.3)	75 (18.3)			
Unskilled manual worker	135 (86.5)	21 (13.5)	156 (39.0)			
Skilled manual worker	39 (75.0)	13 (25.0)	52 (13.0)			
Non-manual worker	54 (64.3)	30 (35.7)	84 (21.0)			
High-level occupations	18 (54.5)	15 (45.5)	33 (8.3)			
Socioeconomic status					<0.001**	69.927**
Low	212 (88.3)	28 (11.7)	240 (60.0)			
Medium	93 (73.8)	33 (26.2)	126 (31.5)			
High	9 (26.5)	25 (73.5)	34 (8.5)			
Total	314 (78.5)	86 (21.5)	400 (100)			

Table 3 shows the family size by family planning history. Around one-third (32%) of the women mentioned that both the husband and wife decided on the desired number of children, while 31.8% of the women mentioned that no one made that decision. The least prevalence of large families (60.9%) was found when both spouses decided on the number of children (p < 0.001).

**Table 3:** Family size by family planning history *Column total was calculated. **p-value was calculated by the Fisher’s exact test. ***p-value was calculated by Pearson Chi-squared test. ****Yes: At least one planned pregnancy. *****No: All unplanned

Variables	Large family size	Small/medium family size	Total	p-value	Statistical test value
	No. (%)	No. (%)	No. (%) *		
Who decided on the number of children				<0.001**	36.785**
Wife	27 (79.4)	7 (20.6)	34 (8.5)		
Husband	80 (87.9)	11 (12.1)	91 (22.8)		
Both	78 (60.9)	50 (39.1)	128 (32.0)		
None	114 (89.8)	13 (10.2)	127 (31.8)		
Mother-in-law	11 (73.3)	4 (26.7)	15 (3.8)		
Others	4 (80.0)	1 (20.0)	5 (1.3)		
Preferred family size				<0.001***	26.293***
Prefer small and medium sized family	144 (69.2)	64 (30.8)	208 (52.0)		
Prefer large sized family	106 (84.1)	20 (15.9)	126 (31.5)		
No idea	64 (97.0)	2 (3.0)	66 (16.5)		
Short birth spacing interval				<0.001***	65.006***
Yes	253 (89.1)	31 (10.9)	284 (71.0)		
No	61 (52.6)	55 (47.4)	116 (29.0)		
Traditional, modern or both methods				0.131***	6.628***
Only traditional contraceptive method	65 (76.5)	20 (23.5)	85 (21.3)		
Only modern contraceptive method	57 (89.1)	7 (10.9)	64 (16.0)		
Combination of both methods	158 (77.5)	46 (22.5)	204 (51.0)		
None of the methods	34 (72.3)	13 (27.7)	47 (11.8)		
Planned pregnancies				<0.001***	44.166***
Yes****	125 (64.4)	69 (35.6)	194 (48.5)		
No*****	189 (91.7)	17 (8.3)	206 (51.5)		
Total	314 (78.5)	86 (21.5)	400 (100)		

More than half (52.0%) of the women preferred small-/medium-sized families, among which 69.2% had large families. On the other hand, the ones who preferred large families, 84.1% of them had large families (p < 0.001). The average preferred number of children to the mothers was 3.4.

The majority (71.0%) of participants had short birth intervals (less than two years) between their live births. The prevalence among women with short birth spacing (89.1%) was significantly (p < 0.001) higher than the prevalence among women with long birth spacing (52.6%). More than half (51.0%) of the women have used both traditional and modern contraceptive methods. There was no significant (p = 0.131) association between the method of contraception used and the family size.

More than half (51.5%) of the participants were those whose pregnancies were unplanned, while 91.7% of those participants had a large size family. Moreover, having at least one planned pregnancy was associated with a lesser prevalence (64.4%) of large-size families (Table 3).

Table 4 shows the obstetric history of the respondents. The mean (SD) gravida and para of the participants were 8.4 (3.2) pregnancies and 7.6 (2.7) deliveries, respectively. The majority (357/400 = 89.3%) of the women were grand multigravida and 95.8% of the pregnancies belonged to this group; the figures were similar for the grand multipara as well.

**Table 4:** Past obstetric history of the sample *Among participating women (n = 400). **Among all pregnancies (n = 3345)

Variables	Frequency	Percentage (%)
Gravidity (n = 3345)		
Primigravida (1/400) *	1	0.03
Multigravida (42/400) *	138	4.13
Grand multigravida (357/400) *	3206	95.8
Mean (SD)	8.4	3.2
Parity (n = 2902)		
Primipara (2/400) *	2	0.07
Multipara (69/400) *	237	8.2
Grand multipara (329/400) *	2663	91.7
Mean (SD)	7.6	(2.7)
Miscarriages	436	13.0 **
Mean (SD)*	1.1	(1.4)
Mode of delivery		
Vaginal delivery	2691	92.7
Cesarean section delivery	211	7.3
Place of delivery		
Home delivery	1747	60.2
Delivery in a health facility	1113	38.4
Delivery in birth attendants' place	42	1.4
Birth spacing interval (live births)		
Birth interval <2 years	1078	46.6
Birth interval ≥2 years	1029	44.5
Birth interval >5 years	207	8.9
Planned or unplanned pregnancy		
Planned pregnancies	595	17.8
Unplanned pregnancies	2750	82.2

Regarding the mode of delivery, 92.7% of all births were delivered vaginally, while only 7.3% were delivered by Cesarean section (CS). In accordance to that, for the place of delivery, home delivery predominated (60.2%) over the health facility delivery (38.4%). Less than half (46.6%) of the live births were delivered with a preceding birth interval of less than two years (short birth spacing interval). The majority (82.2%) of the pregnancies were unplanned, and only 17.8% were planned (Table 4).

Table 5 is the logistic regression analysis table that shows the relationship between the large family size (dependent variable) and several covariates. The factors having statistically significant (p < 0.001) association with the large family size were short birth spacing (odds ratio (OR): 5.841; 95% confidence interval (CI): 3.101-11.002), unplanned pregnancies (OR: 3.419; 95% CI: 1.716-6.813), and younger age at marriage (<20 years) (OR: 5.133; 95% CI: 2.668-9.877). 

**Table 5:** Logistic regression analysis between large family size as a dependent variable with several covariates *Turkmen, Assyrians, and Chaldeans. **Regression coefficient. †Odds ratio. ††Confidence interval

				95% CI^††^ for OR	
	B**	p	OR^†^	Lower	Upper
Living outside Erbil City	0.372	0.267	1.450	0.753	2.794
Muslims	0.848	0.494	2.335	0.206	26.483
Socioeconomic status		0.208			
Low	0.918	0.107	2.503	0.819	7.648
Medium	0.478	0.387	1.613	0.546	4.760
High (reference)					
Short birth spacing	1.765	< 0.001	5.841	3.101	11.002
Unplanned pregnancies	1.229	< 0.001	3.419	1.716	6.813
Age of wife at marriage (years)		< 0.001			
<20	1.636	< 0.001	5.133	2.668	9.877
≥20 (reference)					
Ethnic group		0.412			
Kurds	0.669	0.419	1.953	0.385	9.900
Arabs	1.320	0.198	3.742	0.503	27.855
Others (reference)*					
Constant	-3.413	0.001	0.033		

## Discussion

This research studied the magnitude of large-sized families and associated factors as well as the past obstetric history among women who had completed their families in Erbil, KRI. This age group (45-74 years) was chosen because, by this age, the probability of giving birth to another child is low [31,32]. So, the family size is completed.

The prevalence of large family size is 78.5% among the studied population, with an average of 8.5 members in each family. Meanwhile, the completed (lifetime) fertility rate of the women is 6.78 live births per woman. This result nearly matches that of the Iraq Family Health Survey (IFHS, 2006/7), which was 6.38 for the age group 45-49 years [16]. These figures are decreasing over time, as a part of the global fertility decline. As in KRI, the average live births for the age group 45-49 years have declined to 5.7 and 4.4 live births per woman in 2011 and 2021, respectively [13].

One of the important associated factors with the large family size is the age of marriage. Marriage for both spouses before 18 years tends to end up with a larger family size. The latter the age at marriage, the smaller the size of the family as also concluded by Prasangi et al. (2021) [33] and Moyo et al. (2022) [34]. The mean age of the respondents at marriage was 19 years (min: 11), and their mean age at the first pregnancy was 19.9 years; these findings are higher than those of the Ertem et al. (2008) study, conducted in Mardin, Turkey, which found to be 16.1 (min: 11) and 17.5 years, respectively [35]. In recent years, the mean age of marriage has been seen to be increasing. According to the KRI Demographic Survey of July 2018, the mean age of KRI women at marriage was 20.7 and that for men was 24.5 [36].

As for religion, our results show that Muslims have larger families than Christians, and this is true globally, mainly due to their higher fertility rates [37]. In this regard, our study findings match the findings of a study conducted in Sri Lanka by Prasangi et al. (2021) [33], who concluded that Muslim families were larger in size than Christian families.

The mean household size in the current study is 7.3 members per household. This figure is declining; according to the Iraqi Women Integrated Social and Health Survey (IWISH1 and IWISH2), it was 5.2 and five members, respectively [13]. Due to the growth in modernization and urbanization of the region, especially in urban areas, the extended family structure shifted toward a more nuclear family structure, even within the large family sizes [38].

One of the cornerstones among all factors affecting women's status in general [39], and fertility and family size in particular, is the educational and occupational levels of the women. A growing number of studies on this subject support the idea that the higher the educational and occupational levels of women, the lower the fertility rates and the smaller the family sizes [33,34].

SES has an inverse relationship (although it is not statistically significant according to the logistic regression analysis) with family size. The lower the SES, the higher the prevalence of a large family size. This finding matches that of Bongaarts (2001); he gave a partial explanation for this finding to be due to the age of marriage [40]. 

When it comes to the decision on the number of children, the prevalence of large family size is lowest when both spouses have decided on the number, and it is the highest when neither of the spouses has decided on the number of their children. The average number of children preferred by the respondents is 3.4, which aligns with 3.6 children in IWISH2 (2022) for the Kurdistan region [13]. This finding is slightly lower than that of Jordan (4.0) and higher than that of Turkiye (2.5) [41].

Regarding the short birth spacing interval, nearly half (46.6%) of the live births were born with an interval of less than two, and the majority (71.0%) of the respondents had at least one short birth interval among their children. This result is a bit higher than that of a local study conducted by Mohammed and Zangana (2022) in Erbil, which was found to be 63.4% [42]. This difference could be due to the age groups included in the studies. Second, changed policies [43], perceptions, and utilization of family planning services over time play a great role [39]. Short birth interval is significantly associated with large family size.

The lifetime contraceptive prevalence (for both methods) of the study population is 88%, and 12% of the women had never used any method of contraception during their entire reproductive years. On the other hand, 66% of the participants had used modern contraceptives at some point in their reproductive lives. However, this figure is much higher when compared to US women, where the majority (90%) have used a modern contraceptive method at some point in their lifetime [44].

Roughly half of the participants did not have any planned pregnancies throughout their reproductive lives; among which the prevalence of large families is 91.7%, compared with 64.4% among the participants who had at least one planned pregnancy. The prevalence of unplanned pregnancies among all pregnancies is 82.2%, which is nearly twice as high as that of the Arab region (40%) [45].

Almost all (99.9%) of the participants are multigravida/grand multigravida (also for multipara/grand multipara), which corresponds to the Iraq Family Health Survey (IFHS) report, 2006 (94.9% were multigravida) [16]. This indicates the high fertility of Iraqi women. The slight variation is for the infertile (who are excluded from the current study) and primigravida women.

The majority (92.7%) of births were by vaginal delivery. The CS rate among all the deliveries was 7.3%; however, the ideal rate is considered to be between 10% and 15%. The continuous raising of the CS rate globally and regionally indicates an increase in unnecessary (not medically indicated) CSs. The rate was 49.1% in Erbil (having the highest rate among all Iraq governorates) according to Multiple Indicator Cluster Surveys 6 (MICS6) [15].

Home delivery predominates (60.2%) over health facility delivery (38.4%) in this study. These figures have changed a lot over time; according to the MICS6, the home delivery percentage was only 14%, and delivery in health facilities has risen to 86% in Erbil in 2018 [15]. This increase in health facility delivery may ensure healthier births for neonates and mothers.

Limitations

One of the limitations of this research is the study setting, which is the Maternity Teaching Hospital. The patients visiting this hospital are usually of low to medium socioeconomic backgrounds since it is a public hospital and provides almost free services. Although nearly all of our participants were the companions and relatives (who came to visit the patients) of the patients who were of more diverse SES, this might have affected the results being representative of the society. Furthermore, choosing a convenience sample as a sampling method also might have affected the generalizability of the findings. Lastly, since the study is about past events, recall bias is another limitation. The respondents might not have remembered the very details of their fertility history.

## Conclusions

In conclusion, the prevalence of large family sizes is high among women who have completed their families in Erbil, KRI. Many demographic and socioeconomic factors are associated with large family sizes, such as rural residency, age <18 at marriage, low SES, short birth spacing interval, and unplanned pregnancies. The majority of women were grand multipara. Mainly, the pregnancies were unplanned, the deliveries were vaginal and at home, and with short birth intervals.

For a more desired family size, it is recommended that young females be empowered by encouraging their education and providing occupation opportunities. In addition to that, prohibiting early marriage and providing/strengthening premarital programs focusing on the importance of family planning, including birth spacing and utilizing contraceptive methods properly, play great roles. We recommend thorough research on the risk factors (medical and nonmedical) associated with the increased rates of CSs nowadays.
